# Community pharmacists’ perception of knowledge, attitudes, and practices toward oral health promotion: A Cross-sectional study on the influence of training, demographics, and resources in the UAE

**DOI:** 10.1371/journal.pone.0323193

**Published:** 2025-06-03

**Authors:** Khalid Awad Al-Kubaisi, Derar H. Abdel-Qader, Nadia Al Mazrouei, Ahmad Al-Azayzih, Semira Abdi Beshir, Asim Ahmed Elnour

**Affiliations:** 1 Department of Pharmacy Practice and Pharmacotherapeutics, College of Pharmacy, University of Sharjah, Sharjah, United Arab Emirates; 2 University of Petra, Amman, Jordan; 3 Department of Clinical Pharmacy, Faculty of Pharmacy, Jordan University of Science and Technology, Irbid, Jordan; 4 Department of Pharmacy Practice, Dubai Pharmacy College for Girls, Dubai, United Arab Emirates; 5 College of Pharmacy, Clinical Pharmacy Program, Al Ain University, Abu Dhabi, United Arab Emirates; Endeavour College of Natural Health, AUSTRALIA

## Abstract

Community pharmacists (CPs) are crucial in promoting public health, including oral health, due to their accessibility and frequent patient interaction. This cross-sectional study examined factors influencing oral health promotion among 350 CPs in the UAE, focusing on training, demographic factors, and resource availability. Bivariate analysis showed that gender, years of experience, and the availability of a consultation area significantly influenced CPs’ confidence in providing oral health advice. Male pharmacists exhibited higher confidence in addressing tobacco-related dental issues compared to females. Those with a consultation area were more likely to perceive oral health promotion as part of their role. Multivariate logistic regression revealed that training significantly predicted engagement in oral health promotion. CPs who received formal training were seven times more likely to promote oral health (OR = 7.260, p < 0.001), while self-training had an even more substantial influence (OR = 39.249, p < 0.01). However, pharmacists who lacked sufficient workflow, staffing, and time were 87% less likely to perceive oral health promotion as part of their role (OR = 0.133, p < 0.001). The proximity of a dental clinic did not significantly affect pharmacists’ involvement in oral health promotion. In conclusion, this study emphasizes the importance of targeted training and adequate resources in encouraging CPs’ participation in oral health promotion. Pharmacists with more experience and access to training are more likely to take on proactive roles in promoting oral health.

## Introduction

Community pharmacists (CPs) are healthcare professionals practicing in community-based pharmacies who provide direct patient care through dispensing medications, counseling on drug usage, managing minor ailments, and offering preventive health advice, including oral health education [1–4] As CPs are the most accessible healthcare providers, often being patients’ first point of contact for minor ailment, [2–11], these professionals are recognized as integral players in promoting public health, providing a wide range of healthcare services that extend beyond traditional medication dispensing [1–4]. As accessible healthcare professionals, CPs are often the first point of contact for patients seeking advice on various health-related issues, including oral health [5–8]. For example, CPs can provide educational resources, offer guidance on proper dental hygiene practices, and recommend over-the-counter products for oral health concerns such as toothaches or gum disease [7,9–11]. They may also refer patients to general dentists or dental specialists for further evaluation or specialized care, depending on patient needs [8–12]. Furthermore, in the UAE, CPs have within their scope of practice a role for early detection and prevention of oral health problems [5,13,14]. Therefore, CPs serve as valuable resources for patients seeking guidance on oral health.

Previous studies in Australia [8,15], the UK ([16,17], Japan [9], Malaysia [7], Nigeria [18], Saudi Arabia [19,20], and Lebanon [6] have highlighted the significant impact of CP s’ involvement in oral health promotion, education, and the provision of oral health products. For instance, in Lebanon [6], a national survey using CP samples demonstrated that CPs’ have confidence in their skills to provide oral health advice, demonstrating good perceived knowledge of this practice area. Moreover, CPs also manifested positive attitudes toward promoting oral health education. In Saudi Arabia, CPs are willing to provide oral health advice but face barriers such as a lack of regular meetings with dental professionals and a lack of oral health knowledge and training. Furthermore, most CPs referred patients requiring dental care from a specialist to the nearest dental clinic, thus enhancing early access to treatment and preventing escalations of oral health problems [20].

Research conducted elsewhere, including Malaysia, Saudi Arabia, Australia, and Lebanon, indicates that training, resources, and demographics significantly impact community pharmacists’ engagement in oral health promotion [1,2,4,15–21]. However, similar studies conducted in the United Arab Emirates (UAE) are lacking. While it is recognised that other potential determinants, such as remuneration, policy environment, or patient perceptions, may play a role in CP oral health promotion, these determinants were beyond the scope of this exploratory study and could be considered in future research.

Therefore, this study aimed to fill this gap by identifying and analyzing the key determinants influencing pharmacists’ perception of oral health promotion as part of their professional role in the UAE. Specifically, our study examined how resource availability, such as workflow and staffing, impacts their willingness and ability to engage in oral health promotion. Additionally, it assessed the role of training in shaping pharmacists’ perceptions and confidence in this area.

## Methods

A cross-sectional study provided a snapshot of the current situation regarding the CPs’ perception of knowledge, as well as their attitudes, and practices toward dental care in the UAE. This design was chosen because it allows for the analysis of relationships between various factors and facilitates the drawing of meaningful conclusions, aligning with the study’s objectives [22]. A cluster sampling method was employed to select a representative sample of CPs. This study was conducted from October 2023 to March 2024 across three emirates: Dubai, Sharjah, and Ajman. A total of 399 community pharmacies were initially selected, with 133 pharmacies from each emirate, resulting in a total sampling frame of 399 pharmacies. An equal number of pharmacies (133 per emirate) from Dubai, Sharjah, and Ajman was selected to ensure sufficient representation from each emirate, facilitating meaningful comparisons across these regions. This approach, although not strictly proportionate to the pharmacist populations in each emirate, was chosen to enhance regional comparability within this exploratory study. The contact details and locations of these pharmacies were obtained from the yellow pages.

To ensure randomness and precision, Microsoft Excel was used to randomly allocate the desired sample size from each emirate. After accounting for potential non-responses, the final sample included 350 community pharmacists, which was determined to be sufficient based on a 95% confidence level and a 5% confidence interval. The minimum sample size required for this study was calculated to be 351 participants, which was justified by the need to achieve a 95% confidence level with a 5% margin of error. This sample size was deemed adequate to provide statistically significant insights into the perceived knowledge, attitudes, and practices of CPs in the UAE regarding dental care.

Only CPs were included in this study. The decision to focus exclusively on CPs was made to align with the research objectives, leverage their accessibility and expertise, and facilitate targeted interventions. This focus also enhances the relevance of the findings for future comparative analyses related to dental care within the community pharmacy setting. Pharmacy assistants, technicians, students, hospital pharmacists, other healthcare professionals, and administrative staff were excluded from this study. The exclusion of these groups was a strategic decision aimed at minimizing potential confounding factors and improving the internal validity of the study’s findings.

### Research instrument

The questionnaire was adapted from a previously validated tools [6,23] to assess perceived knowledge, attitudes and practices of CPs in providing oral health advice. The questionnaire was in English, designed on the Google Forms software platform, and comprises five categories (Appendix). The first category of the assessment is the sociodemographic characteristics of the CPs, incorporating age, gender, nationality (Arab versus non-Arab), job title (pharmacist versus in charge pharmacist), work experience (years), working time (hours), the educational qualification of the CP, the type of pharmacy (Chain/banner group pharmacy versus independent pharmacy), the primary place of practice (shopping center/shopping strip pharmacy, standalone community pharmacy, pharmacy next to medical Center), the number of customers usually interact with daily (<20 customers, 20–30 customers, ≥30 customers), type of customers usually interact with (regular customers, pass by customers, both types of customers), usually serve two customers simultaneously (always, frequently, rarely), if the pharmacy has a private/semi-private consultation area (no private consultation area—no space to create one, semiprivate consultation area, and private consultation area), your practice site has adequate workflow, time, and staff to provide pharmaceutical care service (no staff/no time, yes, workflow/not enough staff, yes, workflow/time/staff), availability of a dental clinic close to the pharmacy (yes, no).

The second category focused on the perceived knowledge in dental care and comprised five questions: (1) the level of perceived knowledge for the most common oral conditions (poor, fair, good, very good, and excellent) (2) the level of training already received in oral health (no training, self-training, conferences & seminars, formal training modules and other (please specify) (3) the level of perceived knowledge confidence when advising on some dental problems such as teething, bad breath, loose crowns, lost dental fillings, trauma to teeth, bleeding gums, gum diseases, dry mouth, sensitive teeth, discolored teeth, denture problems, tobacco-related dental problems, oral ulcer, and oral cancer rated at five Likert scale (very confident, fairly confident, neutral, fairly unconfident, and very unconfident); (4) the interest in receiving further training will be rated at five Likert scales (strongly agree, agree, neutral, disagree, and strongly disagree). and (5) the need for including specific modules related to oral health in the undergraduate pharmacy curriculum rated at four Likert scales (strongly agree, agree, neutral, disagree, and strongly disagree).

The third category focused on the attitude score and was evaluated using three questions to assess pharmacists. (1) are aware of their role in oral health promotion; (2) are willing to engage in a better counseling role in oral health promotion; (3) consider that the dentist- pharmacist collaboration could offer more effective oral health promotion strategies. Each attitude question will be rated on a five-point Likert scales (strongly agree, agree, neutral, disagree, and strongly disagree).

The fourth category concerned the practice of pharmacists related to (1) resources usually used to seek information about oral health (including internet sites, electronic references and databases, books, medical journals, and general dental practitioners (GDPs). The pharmacist gets one point for each correct answer (multiple answers possible).; (2) difficulties in obtaining oral health information rated at 5 Likert scales (strongly agree, agree, neutral, disagree, and strongly disagree). 3) most frequently reported oral conditions requiring counseling include toothache, mouthwash, toothbrush, toothpaste, and bleeding gums; (4) information given to the patient

buying an oral product (should provide information on the proper use of the product, potential side effects, and any drug interactions); (5) dietary advice regarding oral health includes advice on gum problems, denture hygiene, oral cancer, and oral health problems related to cancer and cancer treatments (dry mouth, mucositis, etc.); (6) frequency of providing advice on oral health condition depending on the situation such as toothpaste, mouthwash, cleaning in-between teeth, gum care, denture hygiene, tooth erosion, dry mouth, dietary advice to oral health, smoking cessation, and alcohol consumption (less than once a month, less than once a week, 1–5 times per week, 5–10 times per week, more than ten times per week).

The final section regarding the barriers perceived as preventing community pharmacists from delivering oral health education within the community, including (lack of training, lack of information to give to patients, difficulty deciding when to refer, and limited interaction between dentists and pharmacists). The pharmacist gets one point for each correct answer (multiple answers possible). The final score for each category was calculated by summing the points obtained for all questions.

### Ethical considerations

Ethical approval was obtained from the University of Sharjah’s Research Ethics Committee on June 22, 2023 (Reference number: REC-23-06-21-01-F). Participants were informed about the study’s aim and objectives and provided informed consent before participating. Furthermore, participants were informed that their participation was voluntary, anonymous, and solely for research purposes. They were also aware that they could withdraw at any time before submitting their responses. No personal or health information was collected to ensure privacy.

### Data analysis

Data were analyzed using descriptive statistics to summarize demographic characteristics, perceived knowledge, and CP attitudes regarding oral health promotion. Bivariate analyses, including Chi-square tests, assessed the relationship between gender and confidence levels in providing oral health advice. Logistic regression was used to identify factors influencing CPs’ perception of their role in oral health promotion, focusing on workflow, training, and experience. Odds ratios and p-values were calculated to determine the significance of these associations, providing a clear understanding of the key determinants impacting CPs’ involvement in oral health promotion. A p-value threshold of less than 0.05 was considered statistically significant for all analyses. All data generated and analyzed during this study are included in the supplementary file (S1 File).

## Results

### Demographic characteristics

A total number of 350 participants took part in this study. The age distribution showed that the largest proportion of participants were 31-40 years old (162, 46.3%), followed by 22-30 (148, 42.3%), with only 11.4% of participants being over 40 years old. The gender split was relatively balanced, with 51.7% (n=181) male and 48.3% female (n=169).

Ethnic diversity was present, with 62.3% of participants being(n=132) non-Arab and 37.7% (n=218) being Arab participants. All participants held a Bachelor of Science degree, indicating uniform educational attainment. In addition, work experience varied, with 41.7% of participants (n=146) having 1-5 years of experience, 35.7% (n=125) having 6-10 years of experience, 15.4% (n=54) having 11-15 years of experience, and a smaller group of 18 participants (5.2%) having over 15 years of experience. Just under three-quarters of participants (n=267) were employed in chain pharmacies, while 27.4% (n=96) worked in independent pharmacies.

Pharmacy locations were primarily standalone (n=267, 76.3%), followed by shopping centers (n=58, 16.6%), and a smaller proportion near medical centers (n=25,7.1%). Daily patient interaction was reported, with nearly half of pharmacists (n=172) seeing over 30 patients, 35.1% of pharmacists seeing 20-30 patients, and 15.7% of pharmacists seeing fewer than 20 patients per day.

Customer interaction was diverse, with 46.0% of pharmacists serving both regular and occasional walk-in customers, 37.4% of pharmacists serving primarily pass-by customers, and 16.6% of pharmacists mainly serving regular customers. Most participants (n=307, 87.7%) reported interacting with two patients simultaneously. Nearly three out of four pharmacies (72.9%) had a designated consultation area, while just over one in four (27.1%) did not.

Six in ten (n=210, 60%) participants reported having adequate workflows, sufficient time, and appropriate staffing levels to carry out their responsibilities. However, about one-third (n=131, 37.4%) indicated that while their workflows and time allocations were satisfactory, they encountered challenges due to inadequate staffing. A smaller subset (n= 9, 2.6%) faced a lack of both sufficient staff and time.

Regarding weekly work schedules, over half (n= 204, 58.3%) of the participants worked more than 40 hours per week, while the remaining 41.7% worked less than 40 hours weekly. When questioned about the availability of nearby dental clinics, 44.3% of participants (n= n55) confirmed the presence of a dental clinic nearby, while just over half (n= 195, 55.7%) did not have these resources readily accessible.

Approximately one in six participants (17.7%) reported having no prior training in oral health. In contrast, two-thirds of the participants (66.9%) had engaged in self-training. Additionally, about one in seven participants (14.3%) had attended conferences and seminars related to oral health. Table 1 details the demographic characteristics of the study participants.

**Table 1:** Demographic characteristics of the participants (*N *= 350).

Characteristic	Subcategory	Frequency (%)
**Age (years)**		
	22-30	148 (42.3)
	31-40	162 (46.3)
	>40	40 (11.4)
**Gender**		
	Male	181 (51.7)
	Female	169 (48.3)
**Ethnicity**		
	Arab	132 (37.7)
	Non-Arab	218 (62.3)
**Education**		
	BSC. Degree	350 (100.0)
**Work Experience**		
	1-5 years	146 (41.7)
	6-10 years	125 (35.7)
	11-15 years	54 (15.4)
	>15	25 (7.1)
**Type of pharmacy**		
	Chain pharmacy	254 (72.6)
	Independent pharmacy	96 (27.4)
**Place of the pharmacy**		
	Standalone	267 (76.3)
	Shopping Centre	58 (16.6)
	Next to the Medical Centre	25 (7.1)
**Number of patients per day**		
	<20	55 (15.7)
	20-30	123 (35.1)
	>30	172 (49.1)
**Type of customer**		
	Regular	58 (16.6)
	Occasional walk-in customers	131 (37.4)
	Both	161 (46.0)
**Interact with two patients simultaneously**		
	Yes	307 (87.7)
	No	43 (12.3)
**Consultation area**		
	Yes	255 (72.9)
	No	95 (27.1)
**Adequate workflow, time, and staff**		
	Yes, workflow/time/staff	210 (60.0)
	Yes, workflow/not enough staff	131 (37.4)
	No staff//No time	9 (2.6)
**Working hours weekly**		
	<40	204 (58.3)
	>40	146 (41.7)
**Do you know a dental clinic close to your pharmacy?**		
	Yes	155 (44.3)
	No	195 (55.7)
**Previous Training in oral health**		
	No training	62 (17.7)
	Self-training	234 (66.9)
	Conferences & seminars	50 (14.3)

### Perceived knowledge, attitude, and practice toward oral health promotion

#### Perceived knowledge of oral health.

Among the 350 participants, the majority rated their perceived oral health knowledge as either “Good” (n=145, 41.4%) or “Very Good” (n=162, 46.3%). A smaller proportion considered their knowledge to be “Fair” (27, 7.7%), while only 4.6% rated their knowledge as “Excellent.”

In terms of perceived oral health practice, most participants also rated themselves positively, with 46.6% (n=163) indicating their practice was “Very Good” and 35.7% (n= 125) rating it as “Good.” A smaller number rated their practice as “Fair” (n= 36, 10.3%), and a negligible percentage rated their practice as “Poor” (2, 0.6%). None of the participants rated their practice as “Excellent.” The distribution of participants’ perceived knowledge and practice of oral health advice is summarized in Table 2.

**Table 2:** Distribution of participants’ perceive knowledge and attitude of oral health advice (N = 350).

Characteristic	Frequency (%)
*Perceive Oral Health Knowledge*	
* Fair*	27 (7.7)
* Good*	145 (41.4)
* Very good*	162 (46.3)
* Excellent*	16 (4.6)
*Perceive Oral Health attitude*	
* Fair*	2 (0.6)
* Good*	36 (10.3)
* Very good*	125 (35.7)
* Excellent*	163 (46.6)

#### Preferred resources for seeking oral health information of the participants.

**Fig 1:**
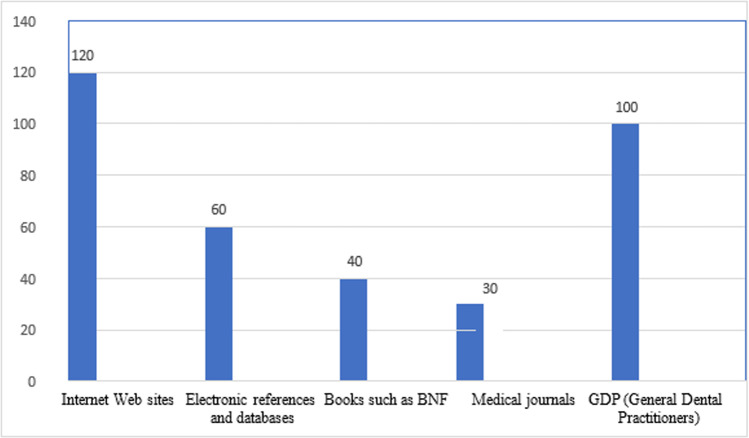
Preferred resources for seeking oral health information among participants.

The survey of 350 participants indicates that the most frequently utilized resources for seeking oral health information are Internet Websites (n=120, 34.3%) and General Dental Practitioners (GDP) (n=100, 28.6%). Other sources include Electronic References and Databases (17.1%), Books such as BNF (11.4%), and Medical Journals (8.6%). **Fig 1** illustrates the distribution of these preferred resources among the participants.

#### Attitude toward dental care.

Approximately three-quarters of participants (77.4%) agree or strongly agree that they would be willing to pursue a more proactive role in oral health promotion, with only a negligible proportion (0.6%) disagreeing.

There is a notable consensus on the value of collaboration between dentists and pharmacists. Herein, 81.1% of respondents either agreed or strongly agreed that collaborations between dentists and pharmacists could offer more effective oral health promotion strategies, and no participants disagreed.

Four out of five participants (79.4%) view oral health promotion as essential to their job as pharmacists, and none of the respondents disagreed. Additionally, while just under three-quarters of participants (72.5%) report routinely giving dietary or other advice to reduce oral side effects due to some drugs, a small percentage (8.0%) disagree or strongly disagree.

A strikingly high percentage (89.1%) of participants expressed interest in receiving further training on oral conditions through continuing professional development courses. Furthermore, most respondents (87.2%) agree or strongly agree that additional oral health advice and training modules should be incorporated into the undergraduate pharmacy curriculum. Table 3 documents the attitude of the participants toward dental care.

**Table 3:** Attitude toward dental care (N = 350).

Attitude	Strongly agree n (%)	Agree n (%)	Neutral n (%)	Disagree n (%)	Strongly disagree n (%)
You would be willing to pursue a more proactive role in oral health promotion.	70 (20.0)	201 (57.4)	77 (22.0)	2 (0.6)	0 (0)
A dentist-pharmacist collaboration could offer more effective oral health promotion strategies.	111 (31.7)	173 (49.4)	66 (18.9)	0 (0)	0 (0)
You perceive oral health promotion as essential to your job as a pharmacist.	99 (28.3)	179 (51.1)	72 (20.6)	0 (0)	0 (0)
You would be willing to pursue a more proactive role in oral health promotion.	98 (28.0)	181 (51.7)	71 (20.3)	0 (0)	0 (0)
You routinely give dietary or other advice to reduce the oral side effects due to some drugs.	82 (23.4)	172 (49.1)	68 (19.4)	18 (5.1)	82 (23.4)
You are interested in receiving further training on oral conditions through continuing professional development courses.	312 (89.1)	0 (0)	0 (0)	0 (0)	312 (89.1)
Additional oral health advice and training modules should be incorporated into the undergraduate pharmacy curriculum.	134 (38.3)	171 (48.9)	43 (12.3)	2 (0.6)	134 (38.3)
You are interested in receiving further training on oral conditions through continuing professional development courses.	312 (89.1)	0 (0)	0 (0)	0 (0)	38 (10.9)
Additional oral health advice and training modules should be incorporated into the undergraduate pharmacy curriculum.	134 (38.3)	171 (48.9)	43 (12.3)	2 (0.6)	0 (0)

#### Priority topics for training programs on oral conditions.

Participant attitudes towards training and improvement are displayed in Table 4. Here, participants prioritize a range of topics for training programs on oral conditions, with gum problems emerging as the most significant topic to be addressed for training, as indicated by nearly a quarter of participants (n= 80, 22.9%).

**Table 4:** Priority topics for training programs on oral conditions of participants (N = 350).

Topics	Frequency (%)
Gum Problems	80 (22.9%)
Smoking Cessation	60 (17.1%)
Prevention of Decay in Children	55 (15.7%)
Dietary Advice in Relation to Oral Health	50 (14.3%)
Directions for the Use of Oral Care Products	40 (11.4%)
Denture Hygiene	30 (8.6%)
Oral Health Problems Related to Cancer and Cancer Treatments	20 (5.7%)
Oral Cancer	15 (4.3%)

The second most relevant topic for training was rated smoking cessation, where one in six participants (n = 60, 17.1%) noted this as important. Preventing tooth decay in children was seen as a priority by 15.7% (n = 55) of the participants. Additionally, in around one in seven participants (n = 50,14.3%), dietary advice about oral health is considered crucial, and directions for using oral care products by slightly over a tenth of participants (n = 40, 11.4%). Denture hygiene is also a priority, as identified by 8.6% of respondents. Further, oral health problems related to cancer and cancer treatments and oral cancer are recognized as essential areas for training by 5.7% and 4.3% of participants, respectively.

#### Practice of oral health promotion.

**Fig 2:**
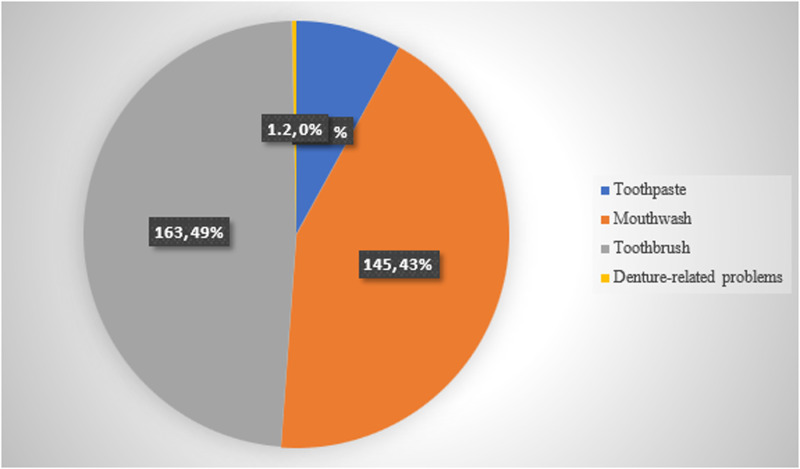
Practice of oral health promotion among participants.

Nearly half of the participants (n=163, 49%) recommended toothbrushes. Mouthwash followed this closely, which 43% (n=145) recommended. Toothpaste recommendations made up a small proportion, at just 3% (n=1), while denture-related problems were the least frequently addressed, with only 0.3% of the recommendations. **Fig 2** illustrates the distribution of recommendations related to oral health products among participants.

#### Bivariate analysis: Confidence levels in providing oral health advice.

Nearly two-thirds of male (62.4%) and female (63.3%) participants reported feeling “Very confident” in providing advice on teething, with no significant difference in confidence levels between genders. Similarly, for bad breath, confidence levels were comparable, with approximately half of the males (about 1 in 2) and slightly more than half of the females (about 5 in 9) indicating they felt “Very confident.” In addition, confidence in advising on loose crowns was lower overall, with approximately 1 in 5 males (19.9%) and about a quarter of females (25.4%) feeling “Very confident.” Similarly, confidence levels for advising on lost dental fillings were relatively low for both genders, with approximately 1 in 6 males (16.6%) and females (16.0%) feeling “Very confident”.

Confidence in handling trauma to teeth was moderate, with similar levels among males (40.3%) and females (40.8%). Likewise, confidence in advising on gum diseases was comparable between genders. More females (45.6%) than males (37.6%) felt “very confident” in providing advice on dry mouth, although this difference was not statistically significant (p<0.05, p=0.643). For sensitive teeth, confidence was high, with 46.4% of males and 50.3% of females feeling “very confident.” Confidence in addressing denture problems was also fairly similar, with 32.6% of males and 27.8% of females reporting high confidence and no significant gender difference (p<0.05, p=0.800).

An exception was found in tobacco-related dental problems, where about one-third of males (35.4%) reported significantly higher confidence than a quarter of females (26.0%), as indicated by a p-value of 0.01. more than half of the participants were “very confident” in advising on oral ulcers (56.9% of males and 51.5% of females), with no significant gender difference. However, confidence was generally low for advising on oral cancer, with only 9.4% of males and 10.1% of females feeling “very confident,” this difference was not statistically significant, as shown in Table 5.

**Table 5:** Assessing confidence levels in providing oral health advice of the participants (N = 350) by gender.

Condition	Very confident	Fairly confident	Neutral	Fairly unconfident	Very unconfident	P value	df	X2
*Teething*								
*Male*	113 (62.4%)	42 (23.2%)	26 (14.4%)	0 (0)	0 (0.0%)	0.35	3	3.24
*Female*	107 (63.3%)	45 (26.6%)	16 (9.5%)	0 (0)	1 (0.6%)			
*Bad breath*								
*Male*	91 (50.3%)	60 (33.1%)	28 (15.5%)	0 (0)	2 (1.1%)	0.82	3	0.912
*Female*	92 (54.4%)	50 (29.6%)	26 (15.4%)	0 (0)	1 (0.6%)			
*Loose crowns*								
*Male*	36 (19.9%)	54 (29.8%)	45 (24.9%)	0 (0)	46 (25.4%)	3.029	3	0.387
*Female*	43 (25.4%)	42 (24.9%)	48 (28.4%)	0 (0)	36 (21.3%)			
*Lost dental fillings*								
*Male*	30 (16.6%)	17 (9.4%)	43 (23.8%)	45 (24.9%)	46 (25.4%)	0.409	4	3.98
*Female*	27 (16.0%)	16 (9.5%)	55 (32.5%)	38 (22.5%)	33 (19.5%)			
*Trauma to teeth*								
*Male*	73 (40.3%)	67 (37.0%)	38 (21.0%)	0 (0)	3 (1.7%)	0.821	3	0.919
*Female*	69 (40.8%)	68 (40.2%)	29 (17.2%)	0 (0)	3 (1.8%)			
*Gum diseases*								
*Male*	73 (40.3%)	66 (36.5%)	39 (21.5%)	0 (0)	3 (1.7%)	0.999	3	0.018
*Female*	69 (40.8%)	61 (36.1%)	36 (21.3%)	0 (0)	3 (1.8%)			
*Dry mouth*								
*Male*	68 (37.6%)	9 (5.0%)	48 (26.5%)	51 (28.2%)	5 (2.8%)	0.643	4	2.511
*Female*	77 (45.6%)	8 (4.7%)	39 (23.1%)	42 (24.9%)	3 (1.8%)			
*Sensitive teeth*								
*Male*	84 (46.4%)	62 (34.3%)	30 (16.6%)	0 (0)	5 (2.8%)	0.641	3	1.684
*Female*	85 (50.3%)	49 (29.0%)	32 (18.9%)	0 (0)	3 (1.8%)			
Denture problems								
*Male*	59 (32.6%)	67 (37.0%)	43 (23.8%)	0 (0)	12 (6.6%)	0.800	3	1.003
*Female*	47 (27.8%)	67 (39.6%)	44 (26.0%)	0 (0)	11 (6.5%)			
*Tobacco- dental problems*								
*Male*	64 (35.4%)	59 (32.6%)	41 (22.7%)	0 (0)	17 (9.4%)	0.01	3	11.134
*Female*	44 (26.0%)	81 (47.9%)	37 (21.9%)	0 (0)	7 (4.1%)			
*Oral ulcer*								
*Male*	103 (56.9%)	50 (27.6%)	18 (9.9%)	0 (0)	10 (5.5%)	0.752	3	1.204
*Female*	87 (51.5%)	55 (32.5%)	17 (10.1%)	0 (0)	10 (5.9%)			
*Oral cancer*								
*Male*	17 (9.4%)	32 (17.7%)	17 (9.4%)	0 (0)	115 (63.5%)	0.882	3	0.661
*Female*	17 (10.1%)	35 (20.7%)	16 (9.5%)	0 (0)	101 (59.8%)			

### Determinants affecting pharmacists’ KAP of oral health promotion

#### Barriers to community pharmacists’ role in oral health education.

Notably, the highest-reported barriers to oral health promotion by pharmacists were reported to be “Lack of Training” and “Limited Interaction Between Dentists and Pharmacists,” each identified by almost one-third (34.3%) of the participants. Additionally, “Lack of Information to Give to Patients” was cited by approximately one-sixth of participants (17.1%). “Difficulty Deciding When to Refer” was reported by 50 participants (14.3%), suggesting challenges in determining appropriate referral situations, as illustrated in Table 6.

**Table 6:** Identifying barriers to participants’ role in oral health education.

Barriers	Frequency (%)
*Lack of training*	120 (34.3%)
*Lack of information to give to patients*	60 (17.1%)
*Difficulty deciding when to refer*	50 (14.3%)
*Limited interaction between dentists and pharmacists*	120 (34.3%)

#### Factors influencing the perception of oral health promotion as part of the pharmacist’s role.

Participants with inadequate workflow, time, and staffing were significantly less likely to view oral health promotion as part of their role (β = -2.017, OR = 0.133), making them about 87% less likely to perceive this responsibility compared to those with adequate resources (p < 0.001).

Furthermore, the absence of a consultation area did not significantly influence the perception of oral health promotion, with a beta coefficient of β =-1.820 and OR=0.162. In addition, participants seeing fewer than 20 patients daily were about 70% less likely to view oral health promotion as part of their role (β = -1.187, OR = 0.305, p = 0.041) compared to those seeing 20- 30 patients daily. Furthermore, participants with over ten years of experience were more than seven times as likely to view oral health promotion as part of their role (β = 1.982, OR = 7.260, p = 0.009) compared to those with less experience. In addition, participants without previous formal training were 38 times less likely to perceive oral health promotion as part of their role than those who had received training (OR = 0.026, 95% CI: 0.002-0.402, p = 0.009).

Interestingly, participants who engaged in self-training were 39 times more likely to view oral health promotion as part of their role compared to those who had not received any training (OR = 39.249, 95% CI: 2.834-543.63, p = 0.006) as illustrated by Table 7.

**Table 7:** Factors influencing the perception of oral health promotion as part of the pharmacist’s role.

Factor	*Response*	*Beta*	*OR*	*95% CI*		*p-value*
Adequate workflow, time, and staff (ref- Yes)	Yes, workflow/not enough staff	−2.017	0.133	0.043	0.416	<0.001
Previous training (ref-Yes)	NO	−3.650	0.026	0.002	0.402	0.009
Previous training (ref-NO)	Self-training	3.670	39.249	2.834	543.63	0.006
*Consultation area * (Ref_Yes)	No	−1.820	0.162	0.040	0.68	0.162
Number of patients daily (ref-20–30)	<20	−1.187	0.305	0.098	0.951	0.041
Experience (Ref_ < 10 years)	>10 years	1.982	7.260	1.635	32.233	0.009

## Discussion

Our study identified six determinants, including resource availability and training, that influence pharmacists in the UAE perceptions of their roles in oral health promotion in the UAE. There was a strong consensus on the importance of collaboration between pharmacists and dentists to enhance oral health promotion, with most pharmacists expressing a readiness to take on a more proactive role in this area. However, our findings also highlight a significant need for targeted training on specific oral health issues, particularly gum problems and smoking cessation programs, to further enhance pharmacists’ effectiveness in promoting oral health.

Our research demonstrates a significant and positive shift in pharmacists’ attitudes towards oral health promotion. The majority of pharmacists are not only willing but also eager to adopt a more proactive role in this area, indicating that pharmacists in the UAE are indeed committed to promoting oral health as suggested by others [1–4]. Furthermore, as in the present study, most participants view oral health promotion as essential to their job, it can therefore be concluded that there is a growing recognition of the importance of integrating oral health into everyday pharmacy practice. Hence, healthcare policymakers could consider expanding the scope of pharmacy practice to formally include oral health promotion, potentially creating guidelines and frameworks that support this integration.

Results in this study also demonstrate a broad agreement among pharmacists in the UAE on the importance of collaboration with dentists to enhance oral health promotion, which aligns with the growing body of literature emphasizing the benefits of interdisciplinary collaboration in healthcare [5–9]. This consensus among pharmacists indicates a recognition of the complementary roles of pharmacists and dental professionals in promoting oral health, therefore contributing to holistic patient care through interdisciplinary efforts. The implications of this finding are significant for developing healthcare models that incorporate collaborative practices. Health systems and policymakers should therefore consider creating frameworks that facilitate closer working relationships between pharmacists and dentists in the UAE. This could include shared care pathways, joint training programs, or co- located services where pharmacists and dentists work together to provide comprehensive care.

Considering these aspects, it can be argued that the results of this study have significant implications for pharmacy practice and education. Firstly, pharmacy schools should consider integrating a more robust oral health training schedule into their undergraduate curricula. This could include dedicated modules on oral health, interdisciplinary training with dental professionals, and practical components that prepare students to address oral health concerns in their future practice. Secondly, professional bodies and continuous education providers should respond to the demand for CPD courses on oral health by developing and offering targeted programs. These courses should be designed to fill the gaps in knowledge that pharmacists perceive, focusing on practical, evidence-based approaches to oral health promotion and patient counseling.

In terms of training for dental problems, pharmacists in the UAE consider they need most training on Gum Problems. This finding aligns with the previous studies [10–14] noting that pharmacists lack confidence in advising customers on these issues. Gum problems, such as gingivitis and periodontitis, are prevalent oral health issues that can lead to severe health complications if not properly managed [10]. Community pharmacists are often the first point of contact within healthcare systems because of their easy accessibility, availability without appointments, and convenient locations, making them a primary resource for initial health consultations, including oral health inquiries [1–4].

In terms of smoking cessation as a resolution to dental issues, one of the most notable findings from the bivariate analysis assessing confidence levels in providing oral health advice by gender is a significant gender difference observed in confidence levels related to advising on tobacco- related dental problems. This difference can be attributed to a variety of factors, including differences in communication experiences [15], perceptions of risk [16], educational background [17], and societal expectations [18]. These factors collectively influence the confidence levels of healthcare providers when advising patients on tobacco cessation, with notable disparities between males and females. This finding suggests that while overall confidence levels are comparable between genders, specific topics such as tobacco-related dental issues may require targeted interventions to address confidence gaps, particularly among female participants.

There is sufficient evidence [11,19,20,22] to demonstrate that there are ongoing efforts made by pharmacists to reduce smoking-related oral and systemic diseases. Several interventions have been used by others to support people quitting smoking. For example, community pharmacists in Belgium used a brief motivational interviewing technique to encourage smoking cessation [20]. Two-thirds of smokers were motivated to quit, and a significant reduction in medication use for cough and inhalers was observed among those who purchased cessation aids. This suggests short- term health benefits and the potential for pharmacists to reach a broad range of smokers [20]. The clear prioritization of gum problems and smoking cessation suggests that training programs should focus heavily on these areas and incorporate evidence-based prevention, detection, and management strategies.

While training emerged as a key determinant, it is crucial to recognize that pharmacist involvement in healthcare extends beyond training alone. Addressing barriers such as inadequate staffing, workflow inefficiencies, and limited interaction with dental professionals should accompany training initiatives [21,24–26]. Integrated interventions that optimize pharmacy workflow, increase staffing levels, and foster interprofessional collaboration between pharmacists in the UAE and dental professionals are essential. For example, integrating oral health training with workflow optimization or co-developing care pathways involving pharmacists and dentists can facilitate the practical implementation of training into daily practice, ensuring sustainable and meaningful engagement of pharmacists in oral healthcare delivery [4,14,26].

While responses provided by pharmacists in the UAE taking part in this study demonstrate that this professional group requires training mostly in relation to advising customers on gum problems and smoking cessation, there have also been serval barriers identified to allowing pharmacists to exercise roles for dental health. Herein, we found that inadequate workflow, staffing, and lack of previous training significantly reduced the likelihood of participants perceiving oral health promotion as part of their role. This finding is consistent with earlier studies in the literature [21,24–28]. For example, a study in England suggests that time management could be challenging when integrating new services like oral health advice into pharmacists’ practice because of increased responsibilities and accountability [26]. These findings might be explained by the fact that pharmacists often face time limitations due to high workloads, which restrict their ability to engage in detailed consultations about oral health. This is particularly problematic in community pharmacies, where pharmacists are often the sole healthcare provider available for consultation [25].

In addition, pharmacists in the UAE are expected to manage a wide range of health issues, and oral health may not be prioritized due to competing demands. This can lead to a reactive rather than proactive approach to oral health advice, where pharmacists only address issues when patients request this help specifically [24]. Even so, many pharmacists lack the necessary training to provide oral health advice confidently. Studies have shown that many pharmacists are not familiar with best practices for managing oral health conditions, which can lead to inadequate patient guidance [27–29].

Hence, there is a need for structural and educational changes to better equip pharmacists in the UAE for these expanded roles. These might include hiring additional pharmacy staff or support personnel to manage routine tasks, allowing pharmacists more time to focus on health promotion activities, including oral health advice. In addition, implementing targeted training modules on oral health within continuing education programs ensures pharmacists are well-prepared to provide accurate and practical advice. Furthermore, more efficient workflows and time management strategies, such as automated systems for routine tasks, should be introduced to free up pharmacists’ time for patient counseling and health promotion.

Additionally, we found that pharmacists in the UAE with over ten years of experience are more than seven times as likely to view oral health promotion as part of their role. This aligns with existing literature emphasizing the influence of experience on professional practice [23,30]. This finding can be explained by pharmacists with more years of experience or those working longer hours tend to have higher perceived knowledge and are more likely to engage in oral health promotion activities. For example, a study among community pharmacists in Lebanon suggested that increased experience in terms of working hours positively influences their ability to promote oral health [23].

Given that experience significantly influences the likelihood of pharmacists engaging in oral health promotion, there is a need for targeted training programs for early-career pharmacists. These programs should focus on building confidence and competence in oral health topics, ensuring that less experienced pharmacists are also prepared to take on these responsibilities. In addition, establishing mentorship opportunities where less experienced pharmacists can learn from their more experienced counterparts could help bridge the knowledge gap. Experienced pharmacists could share practical insights and strategies for integrating oral health promotion into daily practice. Furthermore, for pharmacists with fewer than ten years of experience, CPD programs should emphasize oral health promotion and provide opportunities.

Findings in this study further align with the ongoing global movement towards integrated healthcare systems, wherein CPs are increasingly recognized as important players in MDTs aimed at improving patient healthcare outcomes [8,9]. Internationally, pharmacists’ roles are evolving from traditional medication dispensing to active participation in preventive health services, including oral health promotion and education [1,4,14]. Our findings reinforce evidence from other regions suggesting that CPs are not only well-positioned but also willing to engage in broader health promotion activities when provided with adequate resources and training [21,26,29]. Thus, enhancing pharmacists’ competencies and removing identified barriers, as identified in this study, could effectively contribute to achieving global health objectives, emphasizing preventive care and promoting collaborative practices between healthcare professionals [14,26].

Some contrasting results with other studies have been noted. For example, while in the present study, the absence of a physical place for consultations did not significantly influence the perception of oral health promotion among pharmacists, other studies in the literature emphasize the importance of physical spaces for patient counseling [31–33]. This could be explained by the fact that experienced pharmacists, or those in well-resourced pharmacies, might feel confident in providing oral health advice regardless of the physical setting. Alternatively, it might indicate that other factors, such as workflow, staffing, or personal motivation, play a more critical role in shaping these perceptions than the physical environment itself. While consultation areas can be beneficial, they should not be seen as a prerequisite for engaging in health promotion activities. Therefore, policymakers and pharmacy managers should consider other supportive measures, such as enhancing staffing levels or offering training opportunities, to foster a culture of health promotion within pharmacies.

These recommendations should be considered in light of some study limitations. Firstly, the study relied on self-reported data from pharmacists, which may be subject to response and social desirability biases, as participants might overestimate their confidence or willingness to engage in oral health promotion. Secondly, the cross-sectional design of the survey limits the ability to establish causality between the identified barriers and pharmacists’ involvement in oral health education [34]. Third, the study was conducted within a specific geographic region, the UAE, which may limit the generalizability of the findings to other contexts or countries with different healthcare systems and educational frameworks.

Additionally, the survey did not capture detailed information about the specific types of training or interdisciplinary collaboration that pharmacists had previously experienced, which could have provided more profound insights into the nature and quality of these interventions. Finally, the study did not account for variations in pharmacy practice settings, such as community versus hospital pharmacies, which may influence pharmacists’ opportunities and challenges in engaging in oral health promotion. Future research should address these limitations by incorporating longitudinal designs, exploring diverse geographic regions, and examining the impact of specific educational and collaborative interventions on pharmacists’ roles in oral health promotion.

## Conclusion

This is the first study to identify six determinants influencing oral health promotions among community pharmacists. Our results also showed a strong positive attitude among pharmacists toward oral health promotion and adopting a more proactive role. There was broad agreement among pharmacists on the importance of collaboration with dentists to enhance oral health promotion. Additionally, the absence of a consultation area in community pharmacies was not a decisive factor, indicating that other elements, such as workflow efficiency and staffing, play a more crucial role in shaping pharmacists’ perceptions. Policymakers and pharmacy managers should, therefore, focus on creating supportive environments that encourage proactive oral health. promotion, leveraging structural and educational interventions to enhance the overall effectiveness of pharmacy practice in this area.

## Supporting information

S1 File Raw dataset used for statistical analysis in this study. (XLSX)

## References

[1] Blebil A, Dujaili J, Elkalmi R, Tan HLK, Tai MS, Khan TM. Community pharmacist’s role in providing oral health-care services: findings from Malaysia. J Pharm Bioallied Sci. 2020;12(1):64–71. doi: 10.4103/jpbs.JPBS_152_19 32801602 PMC7397998

[2] Baseer MA, Mehkari MA, Al-Marek FAF, Bajahzar OA. Oral health knowledge, attitude, and self-care practices among pharmacists in Riyadh, Riyadh Province, Saudi Arabia. J Int Soc Prev Community Dent. 2016;6(2):134–41. doi: 10.4103/2231-0762.178739 27114953 PMC4820573

[3] Rajiah K, Ving C. An assessment of pharmacy students’ knowledge, attitude, and practice toward oral health: an exploratory study. J Int Soc Prev Community Dent. 2014;4(4):56. 25452930 10.4103/2231-0762.144601 PMC4247553

[4] Al-Saleh H, Al-Houtan T, Al-Odaill K, Al-Mutairi B, Al-Muaybid M, Al-Falah T, et al. Role of community pharmacists in providing oral health advice in the Eastern province of Saudi Arabia. Saudi Dent J. 2017;29(3):123–8. doi: 10.1016/j.sdentj.2017.03.004 28725130 PMC5502909

[5] Iwata H, Nakamura K, Kobayashi N, Fujimoto K, Hayashi N, Yamaura K. Most dentists approve of oral health check-ups for local residents at community pharmacies and desire collaboration with community pharmacists. Drug Discov Ther. 2022;16(6):309–12. 36529485 10.5582/ddt.2022.01091

[6] Iwata H, Nakamura K, Kobayashi N, Fujimoto K, Hayashi N, Yamaura K. Most dentists approve of oral health check-ups for local residents at community pharmacies and desire collaboration with community pharmacists. Drug Discov Ther. 2022;16(6):2022.01091. 10.5582/ddt.2022.01091 36529485

[7] Zerden L de S, Morris M, Burgess-Flowers J. Oral health and social work integration: advancing social workers’ roles in dental education. Health Soc Work. 2023;48(1):43–53. doi: 10.1093/hsw/hlac038 36511330

[8] Sanders KA, Zerden L de S, Zomorodi M, Ciarrocca K, Schmitz KL. Promoting whole health in the dental setting: steps toward an integrated interprofessional clinical learning environment involving pharmacy, social work, and nursing. Int J Integr Care. 2021;21(4):20. doi: 10.5334/ijic.5814 34824569 PMC8603853

[9] Amien F, Myburgh N, Butler N. Location of community pharmacies and prevalence of oral conditions in the Western Cape province. Health SA Gesondheid. 2013;18(1).

[10] Maunder PEV, Landes DP. An evaluation of the role played by community pharmacies in oral healthcare situated in a primary care trust in the north of England. Br Dent J. 2005;199(4):219–23. doi: 10.1038/sj.bdj.4812614 16127405

[11] Taiwo OO, Panas RM. Evaluation of oral health treatment needs encountered by community pharmacists in Plateau State, Nigeria. J Oral Health Commun Dent. 2018;12(1):1–7. doi: 10.5005/jp-journals-10062-0017

[12] Priya S, Madan Kumar PD, Ramachandran S. Knowledge and attitudes of pharmacists regarding oral health care and oral hygiene products in Chennai city. Indian J Dent Res. 2008;19(2):104–8. doi: 10.4103/0970-9290.40462 18445925

[13] Wiener RC, Waters C, Gaydos MS, Bastin M, Abdulhay N, Bhandari R. Sex gaps in perception of tobacco conversations between adult patients who now smoke cigarettes and oral health care providers: NHANES 2017–2020. J Am Dent Assoc. 2023;154(12):1097–105. 37831025 10.1016/j.adaj.2023.09.004

[14] Medawela R, Ratnayake D, Premathilake L, Jayasinghe R. Attitudes, confidence in practices and perceived barriers towards the promotion of tobacco cessation among clinical dental undergraduates in Sri Lanka. Asian Pac J Cancer Care. 2021;6(2):175–9.

[15] Talluri D, Pachava S, Viswanadh V, Chandu V, Chand S, Rani N. Responsibility of dentist towards tobacco quitting: perceptions of dental students. Popul Med. 2019;1.

[16] Shaheen S, Reddy S, Doshi D, Reddy P, Kulkarni S. Knowledge, attitude and practice regarding tobacco cessation among Indian dentists. Oral Health Prev Dent. 2015;13(5):427–34. 25789360 10.3290/j.ohpd.a33924

[17] Phillips L, Nguyen H, Genge T, Maddigan W. Effectiveness and cost-effectiveness of an intensive and abbreviated individualized smoking cessation program delivered by pharmacists: a pragmatic, mixed-method, randomized trial. Can Pharm J. 2022;155(6):334–44. 10.1177/17151635221128263 PMC9647399 36386606

[18] Vauterin D, Tommelein E, Lahousse L. Smoking cessation encouragement by community pharmacists. Int J Integr Care. 2023;23(S1):372. doi: 10.5334/ijic.icic23481

[19] Gómez Martínez JC, Gaztelurrutia Lavesa L, Mendoza Barbero A, Plaza Zamora J, Lage Piñón M, Aguiló Juanola M, et al. Smoking cessation intervention in the community pharmacy: cost-effectiveness of a non-randomized cluster-controlled trial at 12-months’ follow-up. Res Social Adm Pharm. 2024;20(1):19–27. doi: 10.1016/j.sapharm.2023.09.003 37704533

[20] Taing MW, Ford PJ, Freeman C. Community pharmacy staff needs for the provision of oral health care education and advice in Australia. J Am Pharm Assoc. 2020;60(6):993-1000.e9. 10.1016/j.japh.2020.08.005 32863180

[21] Sturrock A, Preshaw P, Hayes C, Wilkes S. We do not seem to engage with dentists: a qualitative study of primary healthcare staff and patients in the North East of England on the role of pharmacists in oral healthcare. BMJ Open. 2020;10(2):e032261. 10.1136/bmjopen-2019-032261 PMC7050309 32114462

[22] Ho AVL, Lau I, Davidson M, Nimmo A, Croker FA. The role of community pharmacists as oral health advisors in the management of oral effects of asthma medications: an exploratory survey. Int J Pharm Pract. 2024;32(4):280–6. doi: 10.1093/ijpp/riae022 38738298

[23] Dhital R, Sakulwach S, Robert G, Vasilikou C, Sin J. Systematic review on the effects of the physical and social aspects of community pharmacy spaces on service users and staff. Perspect Public Health. 2022;142(2):77–93. doi: 10.1177/17579139221080608 35274562 PMC8918882

[24] Taing M, Choong J, Suppiah V, El-Den S, Park J, McCullough M, et al. A cross-sectional survey exploring Australian pharmacists’ and students’ management of common oral mucosal diseases. Pharmacy. 2023;11(5):139. 37736911 10.3390/pharmacy11050139 PMC10514864

[25] Hu J, McMillan S, El-Den S, O’Reilly C, Collins J, Wheeler A. A scoping review of pharmacy participation in dental and oral health care. Commun Dent Oral Epidemiol. 2021;50(5):339–49. 10.1111/cdoe.12651 33893672

[26] Rajiah K, Lim W, Teoh P, Mas’od M, Lim W, Poh Chou L. Community pharmacists’ knowledge, attitudes and practices towards oral healthcare and its management: a systematic review. Int J Clin Pract. 2021;75(9). 10.1111/ijcp.14096 33619786

[27] Jones A, Sturrock A, Elliott E, Gussy M, Maidment I, Nelson D, et al. Community pharmacists’ perceptions on managing people with oral health problems-a prioritisation survey. J Oral Rehabil. 2024;51(5):851–60. doi: 10.1111/joor.13657 38225810

[28] Hajj A, Hallit S, Azzo C, Abdou F, Akel M, Sacre H, et al. Assessment of knowledge, attitude and practice among community pharmacists towards dental care: a national cross sectional survey. Saudi Pharm J. 2019;27(4):475–83. doi: 10.1016/j.jsps.2019.01.010 31061615 PMC6488811

[29] Gomes ZG, Gomes ZJ, Gomes ZH. The contribution of the physical space in medical space. 2023 [cited 2024 Aug 27]; Available from: https://openaccess.cms-conferences.org/publications/book/978-1-958651-65-0/article/978-1-958651-65-0_16

[30] Sandal LF, Thorlund JB, Moore AJ, Ulrich RS, Dieppe PA, Roos EM. Room for improvement: a randomised controlled trial with nested qualitative interviews on space, place and treatment delivery. Br J Sports Med. 2019;53(6):359–67. [cited 2024 Aug 27]. Available from: https://typeset.io/papers/room-for-improvement-a-random-ised-controlled-trial-with-3ol4hd5fta 28768617 10.1136/bjsports-2016-097448

[31] Mondal H, Mondal S. Social desirability bias: a confounding factor to consider in survey by self-administered questionnaire. Indian J Pharmacol. 2018;50(3):143–4. 30166752 10.4103/ijp.IJP_15_17 PMC6106117

[32] Jelić AG, Tasić L, Škrbić R, Marinković V, Šatara SS, Stojaković N, et al. Pharmacists’ clinical knowledge and practice in the safe use of contraceptives: real knowledge vs. self-perception and the implications. BMC Med Educ. 2021;21(1):430. doi: 10.1186/s12909-021-02864-9 34399761 PMC8365278

[33] Setia MS. Cross-sectional studies. In: The Cambridge Handbook of Research Methods and Statistics for the Social and Behavioral Sciences [Internet]. 2023. pp. 269–91. [cited 2024 Aug 28]. Available from: https://typeset.io/papers/cross-sectional-studies-1zz4u74a

[34] Ray JV. Cross-sectional research. In: The Encyclopedia of Crime and Punishment [Internet]. 2015. pp. 1–5. [cited 2024 Aug 28]. Available from: https://onlinelibrary.wiley.com/doi/abs/10.1002/9781118519639.wbecpx130

